# Supplementary material for: Saccharomyces boulardii (CNCM I-745) improves intestinal damage in sepsis by remodeling intestinal flora structure
Source: Front Cell Infect Microbiol. 2025 Jul 31;15:1602792. doi: 10.3389/fcimb.2025.1602792 (PMC12350329; doi:10.3389/fcimb.2025.1602792)
Supplement: Supplementary file 1 [file DataSheet1.zip › summary/summary/7_Differential_analysis/1_diff_abundance/7_Species/Group.BW0_vs_AW0/Group.BW0_vs_AW0_diff_barplot.pdf]

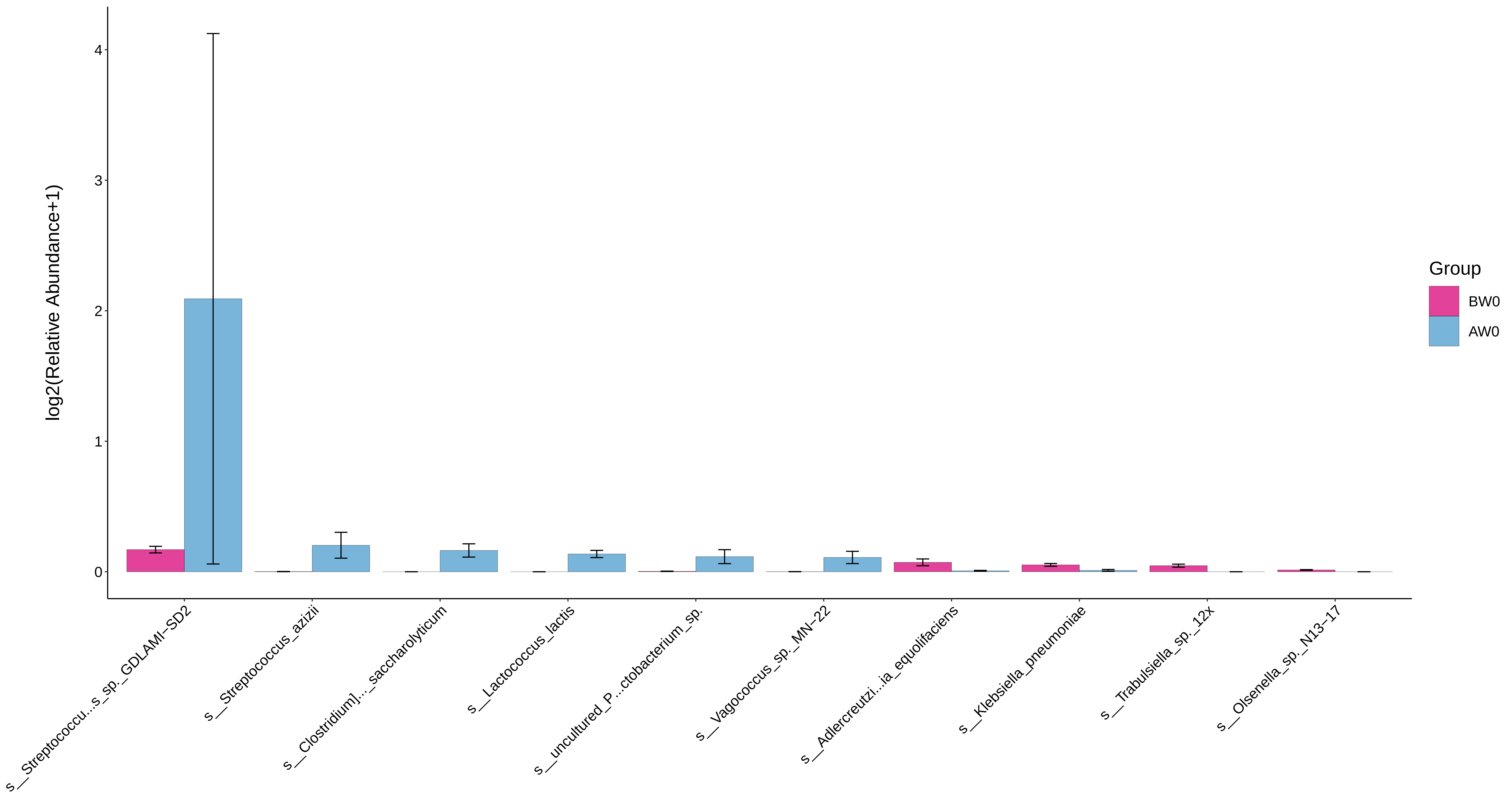

log2(Relative Abundance+1)
0
1
2
3
4
Group
BW0
AW0
s__Streptococcu...s_sp._GDLAMI−SD2
s__Streptococcus_azizii
s__Clostridium]..._saccharolyticum
s__Lactococcus_lactis
s__uncultured_P...ctobacterium_sp.
s__Vagococcus_sp._MN−22
s__Adlercreutzi...ia_equolifaciens
s__Klebsiella_pneumoniae
s__Trabulsiella_sp._12x
s__Olsenella_sp._N13−17
